# Effector Mechanisms in Low-Dose Streptozotocin-induced Diabetes

**DOI:** 10.1155/1998/92198

**Published:** 1998

**Authors:** Miodrag L. Lukić, Stanislava Stošić-Grujičić, Allen Shahin

**Affiliations:** 1 Immunology Unit Faculty of Medicine UAE University P.O. Box 17666 Al Ain United Arab Emirates; 2 Department of Microbiology and Immunology Faculty of Medicine and Institute for Biological Research University of Belgrade Serbia and Montenegro

**Keywords:** Insulin-dependent diabetes mellitus, nitric oxide, RT^6+ T cells, interferon-*γ*, tumor necrosis factor, interleukin- 1, interleukin-1 inhibitor

## Abstract

The cellular and molecular requirements for *β*-cell damages in an immune-mediated toxininduced insulin-dependent diabetes mellitus have been studied in the model of multiple low-dose streptozotocin-induced diabetes in rats and mice. It was found that strain-related susceptibility to diabetes induction correlated with a higher level of IL-2, IFN-*γ*, and TNF-*α* production, whereas such differences were not observed when IL-1 and NO production by macrophages were analyzed; elimination of immunoregulatory RT6^+^T cells that increases IFN-*γ* production, enhances susceptibility to MLD-STZ-induced diabetes; mercury-induced Th-2 cells downregulated the disease; IFN-*γ*-mediated macrophage activation to produce proinflammatory cytokines rather than NO is an important event in early diabetogenic effects of invading macrophages; inhibition of IL-1 activity downregulates diabetes induction; and generation of NO in *β* cells appears to be important for diabetogenic effects. Taken together, data indicate that MLD-STZ diabetes is induced by Th-1 lymphocytes that secrete soluble effector molecules that activate macrophages and promote destruction of *β* cells possibly by both nitric oxide and nonnitric oxide-mediated mechanisms.


Developmental Immunology, 1998, Vol. 6, pp. 119–128
Reprints available directly from the publisher
Photocopying permitted by license only

© 1998 OPA (Overseas Publishers Association)
N.V. Published by license under the
Harwood Academic Publishers imprint,
part of the Gordon and Breach Publishing Group
Printed in Malaysia

### Effector Mechanisms In Low-Dose Streptozotocin-Induced Diabetes

MIODRAG L. LUKIĆ[a, b*], STANISLAVA STOŠIĆ-GRUJIČIĆ[b] and ALLEN SHAHIN[a]

[a]*Immunology Unit, Faculty of Medicine, UAE University, P.O. Box 17666, Al Ain, United Arab Emirates*; [b]*Department of Microbiology and Immunology, Faculty of Medicine and Institute for Biological Research, University of Belgrade, Yugoslavia*

*(In final form 30 May 1997)*

The cellular and molecular requirements for $\beta$-cell damages in an immune-mediated toxin-induced insulin-dependent diabetes mellitus have been studied in the model of multiple low-dose streptozotocin-induced diabetes in rats and mice. It was found that strain-related susceptibility to diabetes induction correlated with a higher level of IL-2, IFN-$\gamma$, and TNF-$\alpha$ production, whereas such differences were not observed when IL-1 and NO production by macrophages were analyzed; elimination of immunoregulatory RT6$^+$T cells that increases IFN-$\gamma$ production, enhances susceptibility to MLD-STZ-induced diabetes; mercury-induced Th-2 cells down-regulated the disease; IFN-$\gamma$-mediated macrophage activation to produce proinflammatory cytokines rather than NO is an important event in early diabetogenic effects of invading macrophages; inhibition of IL-1 activity downregulates diabetes induction; and generation of NO in $\beta$ cells appears to be important for diabetogenic effects. Taken together, data indicate that MLD-STZ diabetes is induced by Th-1 lymphocytes that secrete soluble effector molecules that activate macrophages and promote destruction of $\beta$ cells possibly by both nitric oxide and nonnitric oxide-mediated mechanisms.

*Keywords:* Insulin-dependent diabetes mellitus, nitric oxide, RT6$^+$ T cells, interferon-$\gamma$, tumor necrosis factor, interleukin-1, interleukin-1 inhibitor

### INTRODUCTION

Autoimmune diseases are characterized by a failure of self-tolerance leading the immune system to attack and destroy endogenous tissues. In insulin-dependent diabetes mellitus (IDDM), hyperglycemia is the outcome of a pathological process leading to inflammatory reaction with endothelial-cell activation and accumulation of macrophages, helper, and cytotoxic T cells. The role of T cells in the pathogenesis of IDDM was suggested by (a) their presence in the mononuclear cell infiltrates in the pancreatic islets (Huang et al., 1995), (b) the effects of cyclosporin in delaying disease onset (Bougneres et al., 1988), (c) observation of the transfer of diabetes by transplantation of bone marrow of diabetes patients to an

*Corresponding author. Present address: Immunology Unit, Department of Medical Microbiology, Faculty of Medicine and Health Sciences, UAE University, P.O. Box 17666, Al Ain, United Arab Emirates.

immunodepressed nondiabetic recipient (Lampeter et al, 1993), and (d) the recurrence of insulitis in pancreatic grafts in IDDM patients (Sibley et al., 1985). However, the target of autoimmune response is not fully understood. In fact, the list of candidate antigens is still increasing, including molecules recognized by T cells and those identified by autoantibodies. Most of these autoantigens are not restricted to $\beta$ cells, indicating that microenvironmental factors at the level of the target tissue may be important for the autodestructive process.

### Macrophage Influx Is an Early Event in Diabetes Induction

Macrophages are found to be the first cells to infiltrate the islets in at least two experimental models of IDDM: Multiple low-dose streptozotocin (MLD-STZ) induced diabetes in mice (Kolb-Bachofen et al., 1988) and rats (Lukic et al., 1991) (Figure 1a) and Bio-Breeding (BB) diabetes-prone rats (Hanenberg et al., 1989). Obviously, it is important to distinguish between the role of macrophages as antigen-presenting cells in inducing islet-specific immunity and their role as effector cells directly inducing dysfunction and $\beta$-cell damage. There is ample evidence to support a role of macrophages as effector cells in experimental models of IDDM. Macrophages are cytotoxic to pancreatic $\beta$ cells *in vitro* (Appels et al., 1989). Administration of macrophage toxic agents like silica prevents diabetes in BB rats (Oschilewski et al., 1985). Transfer of diabetes in NOD mice is prevented by blockade of adhesion-promoting receptor on macrophages (Hutchings et al., 1990). Additionally, the finding of heavy macrophage infiltration and acute onset in IDDM patients provides indication for macrophage role in human disease (Lernmark et al., 1995). It was reported that free-oxygen-radical production from activated macrophages is increased in diabetes-prone BB rats before the appearance of inflammatory lesions in the islet (Brenner et al., 1993). It was therefore of interest to study the correlates of macrophage recruitment into the islet during MLD-STZ -induced diabetes.

### IFN-$\gamma$ Enhances Diabetes Induction

Interferon-$\gamma$ plays an essential role in the recruitment of inflammatory cells into delayed-type hypersensitive lesions (Issekutz et al., 1988). Rabinovitch et al. (1996) have compared cytokine gene expression in islet-infiltrating cells of diabetic-prone BB rats, rats protected from diabetes by early treatment with CFA, and diabetes-resistant rats. They found that mRNA expression of T-helper 1 (Th-1)-type cytokines, IFN-$\gamma$, and IL-2 by infiltrating cells correlates with $\beta$-cell-destructive insulitis and IDDM. Furthermore, it was found that destruction of B cells in syngeneic islets transplanted into NOD mice is promoted by cells producing Th-1-type cytokines and prevented by cells producing IL-4 and IL-10 (Suarez-Pinzon et al., 1996).

We studied the susceptibility to MLD-STZ-induced diabetes in two strains of rats, Albino Oxford (AO) and Dark August (DA), which differ in the level of IL-2 (Lukic et al., 1987) and IFN-$\gamma$ production (Arsov et al., 1995). We have previously demonstrated that these differences correlated with the susceptibility to the experimental allergic encephalomyelitis (EAE) (Vukmanovic et al., 1990; Arsov et al, 1995). High IL-2 and IFN-$\gamma$ producer EAE-susceptible DA rats are highly susceptible to MLD-STZ-induced diabetes (Figure 1) and low Th-1 cytokine producer and EAE-resistant AO rats are also resistant to diabetes induction (Lukic et al., 1991; Shahin et al., 1995). This may imply that the level of IFN-$\gamma$ required for optimal stimulation of effector cells in diabetes induction is not achieved in MLD-STZ-treated AO rats. Additionally, there is evidence that exogenous IFN-$\gamma$ may easily enhance diabetes in NOD mice (Campbell et al., 1991). Therefore, we have tested whether the additional availability to IFN-$\gamma$ *in vivo* would overcome the resistance to the induction of MLD-STZ in AO rats. Indeed, when AO rats were given 50,000 i.u. of IFN-$\gamma$ i.v. on days 3, 5, 8, and 10 after the beginning of MLD-STZ regimen (20 mg/kg on days 0 to 4), the resistance to MLD-STZ-induced diabetes was abrogated. It also accelerated the disease development in DA rats (Figures 1b and 1c). Thus, it appears that strain differences in susceptibility to

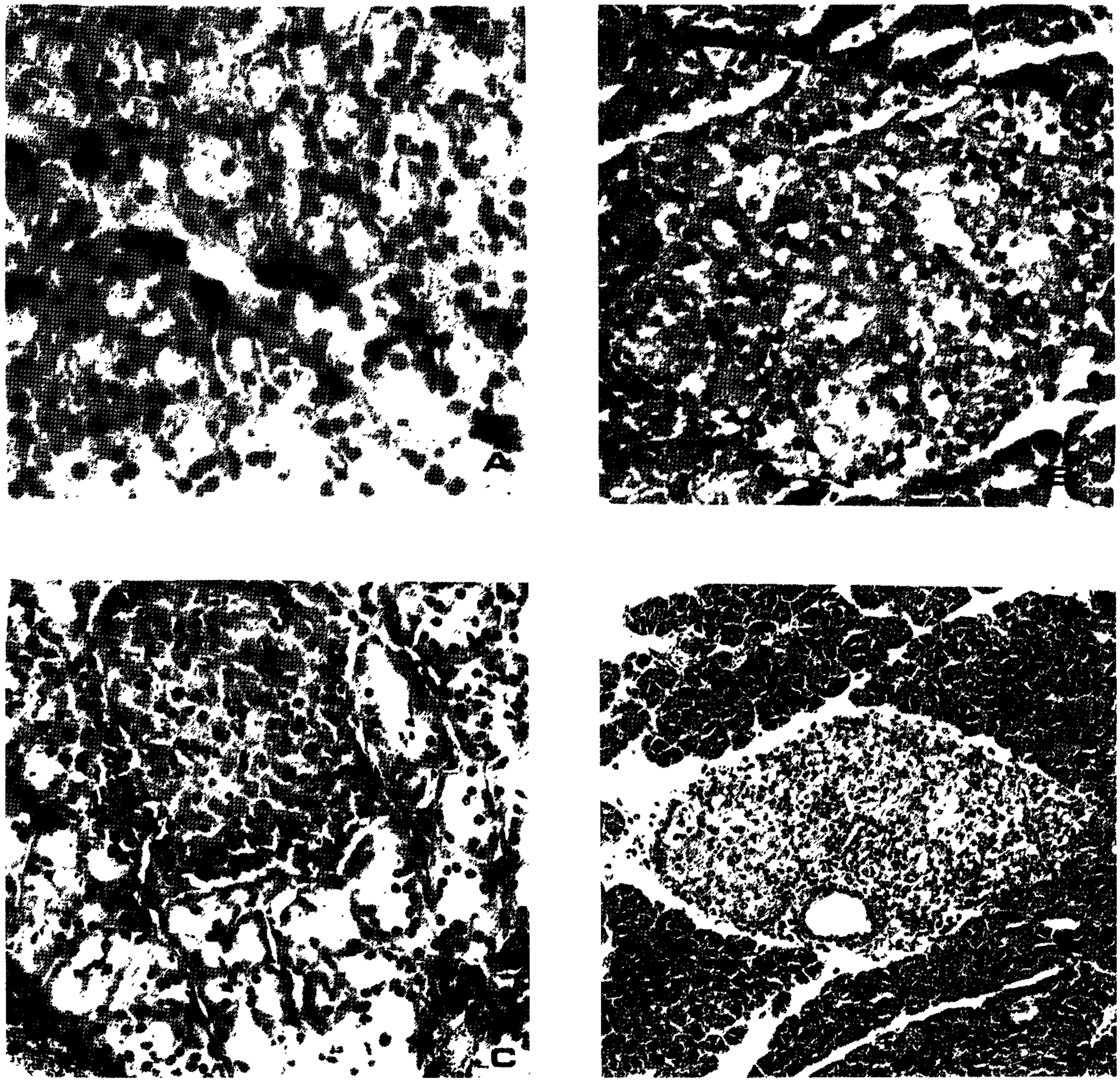

FIGURE 1 MLD-STZ diabetes in susceptible DA rats. (A) Early influx of macrophages: ED-1 positive cells by day 6 after disease induction (PAP). (B) Difuse mononuclear infiltrate in the islet by day 31 after MLD-STZ treatment (HE). (C) $CD4^+$ cells in and around the islet by day 10 after MLD-STZ-diabetes induction (W3/25 mabs, PAP). (D) Mononuclear cell infiltrate in the islets 10 days after inititation of MLD-STZ + IFN-$\gamma$ treatment (see text) (HE).

toxin-triggered autoimmune diabetes does not only correlate with the level of IFN-$\gamma$ production, but may be also manipulated by *in vivo* administration of this cytokine.

It had been shown that diabetes-prone BB rats suffer from a recessively inherited T-cell lymphopenia and failure to express RT6 autoantigen on T cells of lymph node and spleen (Greiner et al., 1987). It appears that RT6 can generate activation signals that result in enhanced expression of a cytokine-receptor molecule (Rigby et al., 1996). We have analyzed the quantitative participation of $RT6^+$ cells in the peripheral T-cell pool of MLD-STZ-induced diabetes-resistant AO and susceptible DA rats. The results clearly indicated that RT6$^+$ cells were significantly more numerous in both CD4$^+$ and CD8$^+$ T-cell subsets in AO rats. To test whether these findings were relevant for the strain-related differences in susceptibility to MLD-STZ-induced diabetes, we pretreated rats with five daily injections (1 ml i.p.) of anti-RT6$^+$ monoclonal antibody containing 6A5 hybridoma culture supernatants prior to the induction of diabetes. The treatment decreased the number of RT6$^+$ T-cells for about 80% in both strains (Pravica et al., 1993). Lymphoid cells derived from AO rats in comparison with DA cells produced significantly less IL-2 and IFN-$\gamma$ in response to Con A stimulation *in vitro* and depletion of RT6$^+$ cells enhanced the level of cytokine production. These increases were fivefold in AO rats for both cytokines. However, although the production of IFN-$\gamma$ was enhanced by anti-RT6 antibody, the level reached after the treatment was three times lower than in normal DA rats. This treatment converted AO rats from resistant to diabetes to mildly diabetic and had a clear enhancing effect in susceptible DA rats.

In studying the role of IFN-$\gamma$ in the induction of MLD-STZ, we attempted to manipulate the susceptibility to the disease by modulating Th-1-Th-2 balance in DA rats. To this end, we used a Th-2-enhancing procedure, the administration of multiple subtoxic doses of mercuric chloride ($HgCl_2$). It had been shown that this treatment may lead in susceptible mouse strains to nonorgan-specific autoimmune disease characterized by increased serum level of IgG1 and IgE antibodies and by the occurrence of antinuclear autoantibodies and immune-complex-mediated glomerulonephritis (Al-Balaghi et al., 1996). It appears that mercury-induced IgG1 and IgE antibody synthesis is T-helper cell-dependent and requires a Th-2-derived cytokine, IL-4 (Al-Balaghi et al., 1996). Pretreatment with $HgCl_2$ protected susceptible rats against experimental autoimmune uveoretinitis induced by immunization with the retinal S antigen. This effect was related to the preferential activation of Th-2 cells and IL-4 production (Saoudi et al., 1993) and indicates that activation of Th-2 cells protects against Th-1-dependent autoimmune disease. Therefore, we have treated MLD-STZ-susceptible DA rats with $HgCl_2$ ($3 \times 1$ mg/kg/day in 2-day intervals) during MLD-STZ-diabetes induction. This treatment had a long lasting protective effect (Figure 2). Depletion of CD8$^+$ T cells *in vivo* did not alter susceptibility to MLD-STZ diabetes in DA rats and protective effect of mercury (Stosic-Grujicic et al., 1996). The results are compatible with the notion that activation of Th-2 cells downregulated MLD-STZ-diabetes induction possibly by affecting the availability of interferon-$\gamma$ at the level of the target tissue.

### Macrophage-Derived Proinflammatory Cytokines Induce $\beta$-Cell Damage

The analysis of the mechanisms by which macrophages elicit its diabetogenic effect is increasingly focused in recent years. Thus, it had been reported that macrophage cytotoxicity toward islet cells *in vitro* is related to macrophage generation of nitric oxide (NO) (Kröncke et al., 1991a). However, it was also shown that macrophage-produced NO is not specifically cytotoxic for $\beta$ cells in the islet (Kröncke et al., 1991b). It was therefore postulated and shown *in vitro* that macrophages exert their effector function via their production of inflammatory cytokines such as interleukin-1 (IL-1) and tumor necrosis factor (TNF). Indeed, several recent studies have demonstrated marked expression of IL-1, TNF-$\alpha$, IFN-$\gamma$, IL-6 RNAs, and/or proteins in the mononuclear infiltrates of islets in NOD mice and BB rats (reviewed in Mandrup-Poulsen, 1996).

*In vitro* studies have also indicated that IL-1 exerts a $\beta$-cell selective toxic effect (Bendtzen et al., 1986). It was thereafter demonstrated that IL-1 reduces $\beta$-cell DNA content (Sandler et al., 1987), induces DNA damage (Delaney et al., 1993), and reduces $\beta$-cell viability (Bolaffi et al., 1994). Although the *in vitro* studies have demonstrated that IL-1 is a most important cytokine mediating $\beta$-cell damage, TNF-$\alpha$ and IFN-$\gamma$ potentiate the action of IL-1 (Eizirik, 1988). Additionally, it had been shown that resident monocytes in normal rat islets stimulated *ex vivo* with LPS will produce sufficient quantity of proinflammatory cytokines to markedly inhibit insulin secretion

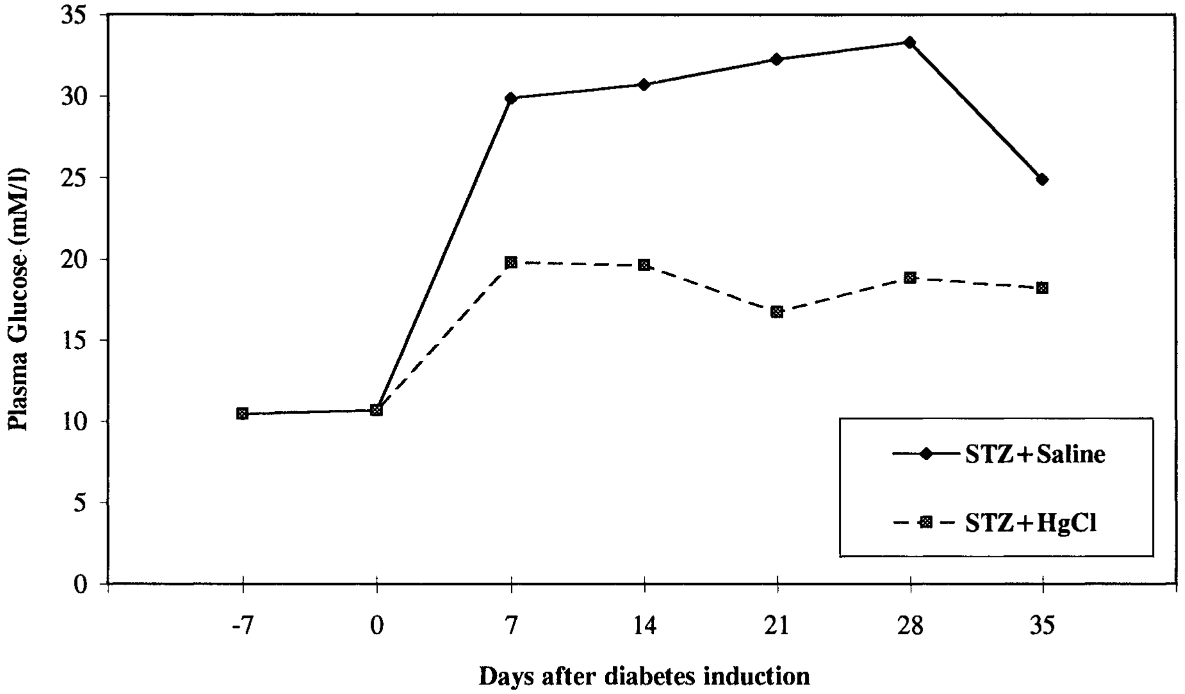

FIGURE 2 MLD-STZ-induced diabetes in mercury-treated rats. Rats were pretreated with $HgCl_2$ (1 mg/kg b.w. in 2-day intervals) from days −6 to 2 in relation to MLD-STZ-diabetes induction (20 mg/kg b.w. for five consecutive days).

(Corbett and McDaniel, 1995). It appears that NO, a free radical formed during the oxidation of L-arginine to L-citrulline, in a reaction catalyzed by inducible NO synthase (iNOS) (Knowles and Moncada, 1994), mediates a number of the effects of IL-1, including the inhibitory effect of IL-1 on $\beta$ cells (Corbett et al, 1991; Delaney et al., 1993). Most recently, Dunger et al. (1996) demonstrated that *in vitro* treatment of islets with TNF-$\alpha$ or IFN-$\gamma$ on their own or in combination inhibited glucose-stimulated insulin secretion in a dose-dependent manner. These effects of TNF-$\alpha$ and IFN-$\gamma$ appeared also to be associated with increased generation of NO. With respect to NO generation, inhibition of glucose-induced insulin secretion and induction of DNA damage in rat islets TNF-$\alpha$ and IFN-$\gamma$ individually exert effects largely similar to those of IL-1.

The validity of the hypothesis that proinflammatory cytokines and nitric oxide generation play a pivotal role in autoimmune-mediated $\beta$-cell damage may be, to a certain extent, tested in the animal models of IDDM. To this end, we have analyzed the effect of TNF-$\alpha$, and IL-1 production and availability in the induction MLD-STZ diabetes in mice and rats. We compared the production of IL-1, TNF-$\alpha$, and NO by activated macrophages derived from rats susceptible or resistant to the induction of diabetes. It was found that recombinant rat IFN-$\gamma$ or LPS + IFN-$\gamma$ stimulation led to severalfold higher production of TNF-$\alpha$ in the cultures derived from DA rats (Table I). These differences were not seen in the production of IL-1 and NO.

### IL-1 Inhibitors Suppress Development of Diabetes

Studies on the impact of systemic IL-1 administration in spontaneous disease models of IDDM have produced different results. The aggravating effects of high doses were observed in BB rats (Wilson et al., 1990) whereas low doses exerted protective effects in NOD mice (Jacob et al., 1990). It should be noted that IL-1 may induce downregulatory cytokine IL-10, which in turn may (Tilag et al., 1995) prevent the

TABLE I Interferon-γ, Interleukin-2, Tumor Necrosis Factor-α and Nitric Oxide Production by Mononuclear Cells of MLD-STZ Diabetes-Susceptible (DA) and -Resistant (AO) Rats

| Producer cells | Mediator | DA | AO |
|---|---|---|---|
| Con A (5 μg/ml) stimulated lymph node cells | IL-2 | 34.36 | 11.57 |
| | IFN-γ | 315.19 | 18.46 |
| Con A (5 μg/ml) stimulated $RT6^-$ lymph node cells | IL-2 | 97.54 | 56.6 |
| | IFN-γ | 302.13 | 97.3 |
| LPS (1 μg/ml) stimulated peritoneal macrophages | TNF-α | 3022.9 | 1431.3 |
| | NO | 51.6 | 65.9 |
| IFN-γ (400 μg/ml) stimulated peritoneal macrophages | TNF-α | 1981.2 | 58.9 |
| | NO | 49.9 | 61.3 |

*Note*: IFN-γ and IL-2 were determined in supernatants of Con A (5 μg/ml) stimulated lymph node cell ($5 \times 10^5$) culture (0.2 ml) at 48 hr. Results are expressed as pg/ml. TNF-α (pg/ml) and NO (μM/l) were determined in supernatants of peritoneal macrophage cultures at 24 hr. of stimulation.

TABLE II Effect of IL-1 Inhibitor on the Development of Insulitis, Islet-Cell Damage, Hyperglycemia, and NO Production

| Animal no. | Treatment | Cellular infiltration score | Islet-cell damage Vacuolization | Islet-cell damage Picnosis | Score | Plasma glucose (mm/l) | Area of iNOS + cells within an islet |
|---|---|---|---|---|---|---|---|
| 1. | STZ + media | + | + | + | + | 17.2 | >50% |
| 2. | STZ + media | ++ | ++ | + | ++ | 22.8 | >50% |
| 3. | STZ + media | ++ | ++ | + | ++ | 19.3 | >50% |
| 4. | STZ + IL-1 INH | + | + | − | − | 8.4 | <5% |
| 5. | STZ + IL-1 INH | + | + | − | − | 9.6 | <5% |
| 6. | ST + IL-1 INH | + | + | − | − | 6.8 | <5% |

*Note*: Based on a minimum of 10 islets per pancreas evaluated mophometrically 25 days after the induction of MLD-STZ diabetes (4 × 40 mg/kg b.w.) in CBA mice. Beginning with the thrid day of STZ injections, mice were given 10 daily i.p. injections of 1 ml of 50-100 kD IL-INH enriched fraction (Stosic and Lukic, 1992) or RPMI-1640. Rabit anti-mouse iNOS was kindly provided by Dr. C. Nathan and applied as described in Stenger et al. (1994).

onset of diabetes, as shown in NOD mice (Pennline et al., 1994). Protection from diabetes with soluble IL-1 receptor was observed in NOD mice (Nicoletti et al., 1994).

We have analyzed the effect of IL-1 inhibition on MLD-STZ-induced diabetes in male CBA mice. Mice injected i.p. with five consecutive daily doses of 40 mg/kg of STZ developed delayed hyperglycemia 10 to 20 days after the completion of the treatment. Sustained hyperglycemia was observed during the next 2 months and mononuclear cell infiltration was present in the islets of the diseased mice. In order to inhibit IL-1 activity during the induction phase of this model, mice received five consecutive days of STZ in conjunction with 10 daily injections of unfractionated rat IL-1 inhibitor (Stosic and Lukic, 1992). Treatment with IL-1 INH started on the third day of STZ and continued until day 12. Elevated levels of glucose in blood in control injected animals were seen by day 10 after completion of STZ treatments and remained high for at least an additional 2 months. This delayed onset of diabetes was characterized with inflammatory infiltrates as well as the vacuolization and picnosis of islet cells (Table II). By contrast, mice receiving STZ and IL-1 INH did not show significant increases in plasma glucose. In agreement with the diabetic status, there was also moderate reduction in the numbers of infiltrating cells and significant decrease in destruction of the islet cells. The effect of rat glucocorticoid-induced IL-1 INH on the onset of diabetes was compared with that of human IL-1Ra. Human IL-1Ra was also protective, although somewhat less than the preparation of IL-1 INH. Partial protective effect of IL-1Ra was observed in a repeated experiment and lasted until the end of the experiments, that is, 7 weeks after the induction of diabetes (Lukic and Stosic, 1993; and to be published). At this time, histologic examination revealed only mild mononuclear infiltration at the periphery of islets in contrast to the widespread insulitis in STZ-treated mice. Thus, both inhibitors of IL-1 activity, that is, rat macrophage-derived glucocorticoid-induced IL-1 INH or human IL-1Ra, diminished the induction of STZ-induced diabetes in mice as evaluated by functional and morphological criteria.

### Nitric Oxide Synthesis and $\beta$-Cell Damage

There is evidence that the deleterious effects of cytokines on islet $\beta$ cells are mediated via induction of NO (Corbett and McDaniel., 1992; Reimers et al., 1996). Inhibitors of NO production protect against IL-1$\beta$-induced $\beta$-cell damage *in vitro* (Bergmann et al., 1992). We (Lukic et al., 1991) and Kolb et al. (1991) have shown that the administration of $N^G$-monomethyl-L-arginine (NMMA), an inhibitor of NO synthesis, modulates the response to the induction of MLD-STZ diabetes in mice. These findings are recently extended by the observations that NMMA significantly reduced the incidence of IDDM in diabetes-prone BB rats (Lindsay et al., 1995; Wu, 1995). However, the source of production of NO during diabetes development was not identified in any of the preceding studies. Early studies have proposed that activated macrophages infiltrating the islets are likely to be an important source of NO (Kroncke et al., 1991b). However, the fact that macrophage cytotoxicity to islet cells via macrophage NO production is not specific for $\beta$ cells (Kröncke et al., 1991a) indicates that macrophages exert their effector function via other soluble mediators, for example, proinflammatory cytokines. Indeed, Southern et al. (1990) have shown that IL-1 with potentiating effects of TNF-$\alpha$ causes NO generation in rat islet cells. An explanation for the $\beta$-cell selective toxicity of IL-1 was provided with the finding of expression of cytokine iNOS, identical to macrophage iNOS in $\beta$ cells (Karlsen et al., 1995) and that this enzyme was not induced in purified non-$\beta$ cells (Strandell et al, 1995). We concluded that the IFN-$\gamma$ + TNF-$\alpha$ + IL-1 *in situ* effect on NO generation in $\beta$ cells (Table II) rather than macrophage-derived NO may be responsible for the higher susceptibility of DA rats to MLD-STZ-induced diabetes.

Finally, it was recently argued that NO production may be neither a necessary nor sufficient precondition for cytokine-induced $\beta$-cell destruction (Mandrup-Poulsen, 1996). For example, although the combination of IL-1$\beta$, TNF-$\alpha$, and IFN-$\gamma$ caused marked NO production in human islets (Corbett et al., 1993), the inhibitors of NO synthase apparently did not prevent the suppressive effects of the cytokines on insulin release and content (Eizirik et al., 1994). Thus, additional mediators or the facilitation of other apoptosis-inducing pathways may be involved. Indeed, Dunger et al. (1996) have found that TNF-$\alpha$ and IFN-$\gamma$ inhibit insulin secretion and cause DNA damage in unweaned-rat islets, but that as cytokine concentrations increase, nonnitric oxide-mediated events predominate. Finally, in an attempt to directly analyze NO involvement in MLD-STZ-induced diabetes, we attempted to induce disease in "knock-out" mice lacking iNOS (Wel et al., 1995). Both (129 × MF1) homozygous mutant mice as well as heterozygous control mice were susceptible to high-dose STZ-induced diabetes but resistant to induction of diabetes with four daily doses of 40 mg STZ. The relative resistance to MLD-STZ-induced diabetes was observed even when five daily injections of STZ were applied. However, at several weeks after the induction of the disease, glycemia was significantly higher in heterozygous than in iNOS-deficient mice (unpublished). The experiments studying the relevance of iNOS deficiency in MLD-STZ-*susceptible* mice should give the final answer concerning the contribution of NO generation in disease induction (our work in progress).

### CONCLUDING COMMENTS

There is a growing body of evidence that Th-1-type $CD4^+$ T cells participate in diabetes induction by producing cytokines that promote destruction of the $\beta$ cells. When this hypothesis is tested in mouse and rat strains susceptible or resistant to induction of MLD-STZ-induced diabetes, the following conclusions could be derived: (1) Strain-related susceptibility to diabetes induction correlated with higher levels of IL-2, IFN-$\gamma$, and TNF-$\alpha$ production, whereas such differences were not observed when IL-1 and NO production by macrophages were analyzed. (2) Elimination of immunoregulatory $RT6^+$ T cells that increases IFN-$\gamma$ production enhances susceptibility to MLD-STZ-induced diabetes. (3) It appears that IFN-$\gamma$-mediated macrophage activation to produce proinflammatory cytokines rather than NO is an important event in early diabetogenic effects of invading macrophages. (4) Inhibition of IL-1 activity downregulates diabetes induction. (5) Generation of NO in $\beta$ cells appears to be important in the development of MLD-STZ-induced diabetes, but the extent of nitric oxide involvement and participation of nonnitric oxide-mediated mechanisms *in vivo* remains to be studied.

### *Acknowledgments*

Secretarial help of Mr. M.V. Raghavan is gratefully acknowledged. Studies were supported in part by an FMHS grant (UAE University) and the Terry Fox Foundation.

### *References*

Al-Balaghi S., Möller E., Möller G., and Abedi-Valugerdi M. (1996). Mercury induces polyclonal B cell activation, autoantibody production and renal immune complex deposits in young (NZB × NSW) F1 hybrids. Eur. J. Immunol. **26**: 1519-1526.

Appels B., Burkart V., Kantwerk-Funke G., Funda J., Kolb-Bachofen V., and Kolb H. (1989). Spontaneous cytotoxicity of macrophages against pancreatic islet cells. J. Immunol. **142**: 3803-3808.

Arsov I., Pravica V., Ejdus L., Badovinac M., and Lukic M.L. (1995). Selection for the susceptibility to experimental allergic encephalomyelitis also selects for high IFN-$\gamma$ production. Transplant. Proc. **27**: 1537-1538.

Bendtzen K., Mandruop-Poulsen T., Nerup J., Nielsen J.H., Dinarello C.A., and Svenson M. (1986). Cytotoxicity of human pI 7 interleukin-1 for pancreatic islets of Langerhans. Science **232**: 1545-1547.

Bergmann L., Kröncke K.D., Suschek C., Kolb H., and Kolb-Bachofen V. (1992). Cytotoxic action of IL-1 against pancreatic islets is mediated via nitric oxide formation and is inhibited by $N^G$-monomethyl-L-arginine. FEBS Lett. **299**: 103-106.

Bolaffi J.L., Rodd G.G., Wang J., and Grodsky G.M. (1994). Interrelationship of changes in islet nicotine adeninedinucleotide, insulin secretion, and cell viability induced by interleukin-$\beta$. Endocrinology **134**: 537-542.

Bougneres P.F., Carel J.C., Castano L., Boitard C., Gardin J.P., Landais P., Hors J., Mihatsch M.J., Paillard M., Chaussain J.L., and Bach J.F. (1988). Factors associated with early remission of type I diabetes in children treated with cyclosporin. N. Engl. J. Med. **318**: 663-670.

Brenner H.H., Burkart V., Rothe H., and Kolb H. (1993). Oxygen radical production is increased in macrophages from diabetes prone BB rats. Autoimmunity **15**: 93-98.

Campbell I.L., Kay T.W.H., Oxbrow L., and Harrison L.C. (1991): Essential role for interferon gamma and interleukin 6 in autoimmune insulin-dependent diabetes in NOD/Wehi Mice. J. Clin. Investig. **87**: 739-742.

Corbett J.A., Lancaster J.R., Sweetland M.A., and McDaniel M.L. (1991). Interleukin-1$\beta$-induced formation of EPR-detectable iron nitrosyl complexes in islets of Langerhans. Biol. Chem. **266**: 21351-21354.

Corbett J.A., and McDaniel M.L. (1992). Does nitric oxide mediate autoimmune destruction of $\beta$ cells? Possible therapeutic interventions in IDDM. Diabetes **41**: 897-903.

Corbett J.A., and McDaniel M.L. (1995). Intraislet release of interleukin 1 inhibits $\beta$ cell function by inducing $\beta$ cell expression of inducible nitric oxide synthase. J. Exp. Med. **181**: 559-568.

Corbett J.A., Mikhael A., Shimizu J., Frederick K., Misko T.P., McDaniel M.L., Konagawa O., and Unanue E.R. (1993). Nitric oxide production in islets from nonobese diabetic mice: Aminoguanidine-sensitive and -resistant stages in the immunological diabetic process. Proc. Natl. Acad. Sci. USA **90**: 8992-8995.

Delaney C.A., Green M.H.L., Lowe J.E., and Green I.C. (1993). Endogenous nitric oxide induced by interleukin-$\beta$ in rat islets of Langerhans and HIT-T15 cells causes significant DNA damage as measured by the "cometi" assay. FEBS Lett. **333**: 291-295.

Dunger A., Cunnigham J.M., Delaney C.A., Lowe J.E., Green M.H.L., Bone A.J., and Green I.C. (1996). Tumor necrosis factor-$\alpha$ and interferon-$\gamma$ inhibit insulin secretion and cause DNA damage in unweaned-rat islets. Extent of nitric oxide involvement. Diabetes **45**: 183-189.

Eizirik D.L. (1988). Interleukin-1 induced impairment in pancreatic islet oxidative metabolism of glucose is potentiated by tumor necrosis factor. Acta Endocrinol. **119**: 321-325.

Elzirik D.L., Sandler S., Welsh N., et al. (1994). Cytokines suppress human islet function irrespective of their effects on nitric oxide generation. J. Clin. Invest. **93**: 1968-1974.

Greiner D.Y., Mordes J.P., Handler E.S., Angelilio M., Nakamura N., and Rossini A.A. (1987): Depletion of RT6.1+ lymphocytes induces diabetes in resistant BB/W rats. J. Exp. Med. **166**: 461-469.

Hanenberg H., Kolb-Bachofen V., Kantwerk-Funke G., and Kolb H. (1989). Macrophage infiltration precedes and is a prerequisite for lymphocytic insulitis in pancreatic islets of pre-diabetic BB rats. Diabetologia **32**: 126-134.

Huang X., Yuan J., Goddard A., Foulis A., James R.F.L., Lernmark A., Pulol-Borrell R., Rabinovitch A., Somoza N., and Stewart T.A. (1995). Interferon expression in the pancreas of patients with type I diabetes. Diabetes **44**: 658-664.

Hutchings P., Rosen H., O'Reilly L., Simpson E., Gordon S., and Cooke A. (1990). Transfer of diabetes in mice prevented by blockade of adhesion-promoting receptor on macrophages. Nature **348**: 639-642.

Issekutz T.B., Stoltz J.M., and Van der Meide P. (1988). Lymphocyte recruitment in delayed-typed hypersensitivity. The role of IFN$\gamma$. J. Immunol. **140**: 2989-2993.

Jacob C.O., Aiso S., Michie S.A., McDevitt H.O., and Acha-Orbea H. (1990). Prevention of diabetes in nonobese diabetic mice by tumor necrosis factor (TNF): Similarities between TNF-$\alpha$ and interleukin-1. Proc. Natl. Acad. Sci. USA **87**: 968-972.

Karlsen A.E., Andersen H.U., Vissing H., et al. (1995). Cloning and expression of cytokine-inducible nitric oxide synthase cDNA from rat islets of Langerhans. Diabetes **44**: 753-758.

Knowles R.G., and Moncada S. (1994). Nitric oxide synthases in mammals. Biochem. J. **298**: 249-258.

Kolb H., Kiesel U., Kroncke K.D., and Kolb-Bachofen W. (1991). Suppression of low dose streptozotocin induced diabetes in mice by administration of a nitric oxide synthase inhibitor. Life Sci. **49**: P1213-P1217.

Kolb-Bachofen V., Epstein S., Kiesel U., and Kolb H. (1988). Low dose streptozotocin-induced diabetes in mice. Electron microscopy reveals single-cell insulitis before diabetes onset. Diabetes **37**: 21-27.

Kröncke K., Funda J., Berschick B., and Kolb-Bachofen V. (1991a). Macrophage cytotoxicity towards isolated rat islet cells: Neither lysis nor its protection by nicotinamide are beta-cell specific. Diabetologia **34**: 232-238.

Kröncke K., Kolb-Bachofen V., Berschick B., Burkart V., and Kolb H. (1991b). Activated macrophages kill pancreatic syngeneic islet cells via arginine-dependent nitric oxide generation. Biochem. Biophys. Res. Commun. **175**: 752-758.

Lampeter E.F., Homerg M., Quabeck K., Schaefer U.W., Wernet P., Bertrams J., Crosswilde H., Gries F.A., and Kolb H. (1993). Transfer of insulin-dependent diabetes between HLA-identical siblings by bone marrow transplantation. Lancet **341**: 1243-1244.

Lernmark A., Kloppel G., and Stenger D. (1995). Heterogeneity of islet pathology in two infants with recent onset diabetes mellitus. Virchows Arch. **425**: 631-640.

Lindsay R., Smith W., Rossiter S.P., McIntyre M.A., Williams B.C., and Baird J.D. (1995). $N^w$-nitro-L-arginine methyl ester reduces the incidence of IDDM in BB/E rats. Diabetes **44**: 365-368.

Lukic M.L., Al-Sharif R., Mostarica M., Bahr G., and Behbehani K. (1991). Immunological basis of the strain differences in susceptibility to low-dose streptozotocin-induced diabetes in rats. In Lymphatic Tissues and *In vivo* Immune Responses, Imhof, et. al., Eds. (New York: Marcel Dekker), pp. 643-647.

Lukic M.L., Mostarica Stojkovic M., Kostic M., Tucic N., and Vukmanovic S. (1987). Cellular and genetic basis of the strain differences in IL2 production in rats. Transpl. Proc. **19**: 3137-3139.

Lukic M.L., and Stosic S. (1993). Interleukin 1 receptor antagonists prevent the induction of autoimmune diabetes. Autoimmunity **14 (Suppl. 1)**: 12.

Lukic M.L., Stosic-Grujicic S., Ostojic N., Chan W.L., and Liew F.Y. (1991). Inihibition of nitric oxide generation affects the induction of diabetes by streptozotocin in mice Biochem. Biophys. Res. Commun. **178**: 913-920.

Mandrup-Poulsen T. (1996). The role of interleukin-1 in the pathogenesis of IDDM (Review). Diabetologia **39**: 1005-1029.

Nicoletti F., Di Marco R., Barcellini W., et al. (1994). Protection from experimental autoimmune diabetes in the nonobese diabetic mouse with soluble interleukin-1 receptor. Eur. J. Immunol. **24**: 1843-1847.

Oschilewski U., Kiesel U., and Kolb H. (1985). Administration of silica prevents diabetes in BB-rats. Diabetes **34**: 197-199.

Pennline K.J., Rouque-Gaffney E., and Monahan M. (1994). Recombinant human IL-10 prevents the onset of diabetes in the nonobese diabetic mouse. Clin. Immunol. Immunopathol. **71**: 169-175.

Pravica V., Ejdus L., Mostarica Stojkovic M., Stosic-Grujicic S., and Lukic M.L. (1993). Resistance to multiple low dose streptozotocin-induced diabetes in rats: Dependence on RT6+ T cells. Transplant. Proc. **25**: 2835-2836.

Rabinovitch A., Suarez-Pinzon W., El-Sheikh A., Sorensen O., and Power R.F. (1996). Cytokine gene expression in pancreatic islet-infiltrating leukocytes of BB rats. Expression of Thl cytolines correlates with $\beta$-cell destructive insulitis and IDDM. Diabetes **45**: 749-754.

Reimers J.I., Andersen H.U., Mauricio D., Pociot F., Karlsen A.E., Petersen J.S., Mandrup-Poulsen T., and Nerup J. (1996). Diabetes **45**: 771-778.

Rigby M.R., Bortell R., Greiner D.L., Czech M.P., Klarlund J.K., and Mordes J.P. (1996). The rat T-cell surface protein RT6 is associated with *src* family tyrosine kinases and generates an activation signal. Diabetes **45**: 1419-1426.

Sandler S., Andersson A., and Hellerström C. (1987). Inhibitory effects of interleukin 1 on insulin secretion, insulin biosynthesis, and oxidative metabolism of isolated rat pancreatic islets. Endocrinology **121**: 1424-1431.

Saoudi A., Kuhn J., Huygen K., de Kozak Y., Velu T., Goldman M., Druet P., and Bellon B. (1993). TH2 activated cells prevent experimental autoimmune uveoretinitis, a TH1-dependent autoimmune disease. Eur. J. Immunol. **23**: 3096-3103.

Shahin A., Mahmoud T.A.M., and Lukic M.L. (1995). Transforming growth factor $\beta$ and inteferon gamma modulate the development of Th-1 mediated autoimmunity in susceptible and resistant strains of rats. Transplant. Proc. **27**: 1535-1536.

Sibley R., Sutherland D.E.R., Goetz F., and Michael A.F. (1985). Recurrent diabetes mellitus in the pancreas iso- and allograft: A light and electron microscopic and immunohistochemical analysis of four cases. Lab. Invest. **53**: 132-144.

Southern C., Schulster D., and Green I.C. (1990). Inhibition of insulin secretion by interleukin-1b and tumour necrosis factor-$\alpha$ via an L-arginine-dependent nitric oxide generating mechanism. FEBS Lett. **276**: 42-44.

Stenger S., Thüring H., Röllinghoff M., and Bogdan C. (1994). Tissue expression of inducible nitric oxide synthase is closely associated with resistance to *lieshmania major*. J. Exp. Med. **180**: 783-793

Stosic-Grujicic S., and Lukic M.L. (1992). Glucocorticoid-induced keratinocyte-derived interleukin-1 receptor antagonist(s). Immunology **75**: 293-298.

Stosic-Grujicic S., Mijatovic S., Ejdus L. and Lukic M.L. (1996). Enhancement of Th2-type activity downregulated diabetes induction. Transplant. Proc. **28(6)**: 3259.

Strandell E., Buschard K., Saldeen J., and Welsh N. (1995). Interleukin-1$\beta$ induces the expression of HSP70, heme oxygenase and Min-SOD in FACS-purified rat islet $\beta$-cells, but not in $\alpha$-cells. Immunol. Lett. **48**: 145-148.

Suarez-Pinzon W., Rajotte R.V., Mosmann T.R., and Rabinovitch A. (1996). Both $CD4^+$ and $CD8^+$ T-Cells in syngeneic islet grafts in NOD mice produce Interferon-$\gamma$ during $\beta$ cell destruction. Diabetes **4**: 1350-1357.

Tilag H., Atkins M.B., Dinarello C.A., and Mier J.W. (1995). Induction of circulating interleukin 10 by interleukin 1 and interleukin 2, but not interleukin 6 immunotherapy. Cytokine **7**: 734-739.

Vukmanovic S., Mostarica Stojkovic M., Zalud I., Ramic Z., and Lukic M.L. (1990). Analysis of T cell subsets after induction of experimental autoimmune encephalomyelitis in susceptible and resistant strains of rats. J. Neuroimmunol. **27**: 63-69.

Wel X., Charles I.G., Smith A., Ure J., Feng G., Huang F., Xu D., Muller W., Moncada S., and Liew F.Y. (1995). Altered immune responses in mice lacking inducible nitric oxide synthase. Nature **375**: 408-410.

Wilson C.A., Jacobs C., Baker P., Baskin D.G., Dower S., Lernmark A., et al. (1990). IL-1$\beta$ modulation of spontaneous autoimmune diabetes and thyroiditis in the BB rat. J. Immunol. **144**: 3784-3788.

Wu G. (1995). Nitric oxide synthesis and the effect of aminoguanidine and $N^G$-monomethyl-L-arginine on the onset of diabetes in the spontaneously diabetic BB rat. Diabetes **44**: 360-364.

